# Medical unfitness for work at sea: causes and incidence rate over a 12-year period in France

**DOI:** 10.1186/s12995-021-00291-6

**Published:** 2021-01-21

**Authors:** Brice Loddé, Marie-Fleur Megard, Nicolas Le Goff, Laurent Misery, Richard Pougnet, Jean-Dominique Dewitte, David Lucas, Thierry Sauvage

**Affiliations:** 1 Université de Bretagne Occidentale, ORPHY, EA 4324, Avenue Le Gorgeu, CS 93837, 29238 Brest Cedex 3, France; 2 Société Française de Médecine Maritime, Brest, France; 3 Service de Santé des Gens de Mer, Brest, France

**Keywords:** Unfitness, Incidence, Maritime, Seafarer, Musculoskeletal disorder, Accident, Mental, Cardiovascular, Neurological, Disease, Incapacity

## Abstract

**Background:**

The purposes of the study were first to determine the incidence rate of medical unfitness for work at sea among French seafarers, second to identify the conditions (diseases or accidents) causing such incapacity so as to set up prevention measures where possible and third to ascertain whether there were any overrepresentations of diseases according to category of unfit seafarers (fishers, merchant seafarers, shellfish farmers and professional sailors).

**Methods:**

An exhaustive, observational, descriptive, retrospective epidemiological and nosological study was carried out based on the medical coding of files stored in the Aesculapius® national database, which registers all medical data regarding seafarers presenting at the French seafarers’ health services. The increasing rate of permanent medical unfitness for work at sea was calculated in relation to the annual number of registered seafarers. A 12-year span was chosen in an attempt to ascertain the different sociodemographic categories associated with incapacity.

**Results:**

In all, 2392 seafarers were declared unfit for work at sea. This represents a permanent medical unfitness for work at sea incidence rate of below 1% for all French seafarers examined for medical fitness between 2005 and 2016.

The average age of the population of unfit seafarers was 48. The average time spent at sea before being declared unfit for work at sea was 15.5 years. Sixty-seven percent of the seafarers declared unfit had been working in the fishing sector.

The main reasons for deciding permanent unfitness for work at sea were: rheumatological conditions associated specifically with the spine; injuries relating to accidents or other external causes, mostly affecting the upper limbs; mental and behavioural disorders, including mood disorders and particularly addictions; and diseases of the circulatory system, namely coronopathies. The incidence rate of medical unfitness for work at sea was seen to increase between 2005 and 2016, but a decrease due to the dilution effect was noted in 2015.

**Conclusions:**

Permanent unfitness seldom occurs among French professional seafarers. Prevention measures must be focused on musculoskeletal disorders, psychiatric affections and coronary conditions as well as on combatting maritime accidents, especially in the professional fishing sector, where such affections and accidents are overrepresented.

## Introduction

In France, as in other littoral countries such as the UK, Poland, the Netherlands, Benin and the Scandinavian countries, any individual working as a professional civilian seafarer must pass a medical fitness examination. Three types of medical examination are obligatory in France: a pre-employment, a return-to-work (after sick leave) and a periodic medical examination (every one to two years depending on the specific case). The Service de Santé des Gens de Mer (SSGM [France’s national seafarers’ health service]) is the government body responsible for determining applicants’ medical fitness for work at sea. During this medical fitness examination, seafarers’ doctors (SDs) (who belong to the SSGM) make their decision based on two fundamental criteria. The first is an assessment of the professional’s health status. The second is an appraisal of their health status in relation to the requirements of their onboard workspace, taking into account the fact that the sea is a hostile environment for humans and that seafarers are (sometimes extremely) remote from land-based health facilities [1]. To summarise, professional seafarers are declared medically unfit for work at sea when their work risks a deterioration in their health status or when their health status puts a strain on onboard community life to the point of endangering the rest of the crew. In France, the SDs are general practitioners or occupational health practitioners who have been certified by the French government. Success in the *Diplôme Universitaire de Médecine Maritime* (the French University Diploma of Maritime Medicine) is required to obtain this certification. The SDs consult the law to guide their assessments. The most recent law passed was the decree of 3 August 2017 on the health and medical fitness requirements for civilian seafarers for work at sea. This derived from the decree of 16 April 1986 amended by the decrees of 27 April 1990, 11 January 1991 and 6 July 2000 [2]. As is the case in other countries, workstations on board French boats vary depending on the category of the vessel. The captain is the boat’s highest-ranking officer and represents the first crew member employed at sea. The rest of the crew may be composed of, for example, engineering officers, mates, fishers and head cooks. French seafarers can be employed on container ships, tanker ships, bulk carriers, passenger ships, offshore vessels, fishing vessels, sailboats and speciality vessels, that is to say civilian ships. Depending on the category of the vessel, work at sea can involve operations anywhere from the territorial seas to the high seas. Deciding that a seafarer is medically unfit for work at sea has serious consequences for the applicant because they will no longer be able to practise their profession and will be forced to retrain. This is therefore a difficult decision for a SD to make. Every SD decision of unfitness or fitness with restrictions is referred on appeal to a medical committee (which, at the time of the study, was called the CMRA, or Commission Médicale Régionale d’Aptitude [regional committee for assessing medical fitness for work at sea] but which is now called medical college, in French *collège médical maritime*), which rules on the finality of the SD’s decision. The four CMRAs in France were each made up of a SD who had not taken part in the initial medical decision, a director and a group of medical experts.

While there are a relatively large number of descriptive studies in the literature on the reasons for medical unfitness in land-based occupational health, there has been no French study conducted to date on the causes of medical unfitness among professional civilian seafarers or on the epidemiology of this population. In almost all non-maritime sectors [3–5], the primary causes of permanent medical unfitness for work are Musculoskeletal Disorders (MSDs) with psychological disorders ranking second.

At international level, there have been very few nosological studies conducted on medical unfitness in the professional maritime environment. In 2014, Zevallos and coll [6] found that approximately 1% of the total seafarer fitness to work assessments conducted in the Netherlands over one year resulted in an unfitness decision. Similarly, in 2019, Ayelo and coll investigated the heath status of a sample of Beninian seafarers presenting for the medical examination for fitness for work at sea and found only a very small proportion of seafarers had been declared unfit for work at sea [7]. Other studies have focused on either case reports [8, 9] or nosological groups, such as cardiovascular diseases [10, 11] and metabolic disorders [12]. However, there has been no study dealing with the main affections at the origin of unfitness decisions and the incidence rate of permanent unfitness for work at sea. The low number of studies generally on medical unfitness among professional seafarers can largely be explained by the difficulty of obtaining large-scale or exhaustive quantitative data, not only on the diseases encountered during this type of medical examination but also on large workforce populations. The maritime sector often employs only a minority of a country’s professionally active population (in France, it employs approximately 0.1% of the total number of workers). We should also add that not all professional seafarers worldwide are subject to standardised medical monitoring that can lead to a decision on fitness or unfitness [13, 14]. This therefore minimises the likelihood of ever seeing epidemiological studies on the nosology of unfitness in this type of population [15]. Nevertheless, this population is associated with medical issues that need to be explored. Working at sea is a difficult job that is associated with a high accident rate [16] (in Japan, the accident rate for seafarers is 5 times higher than for any other industrial sector) [17]. Many individuals working in these professions do not have the capacity to return to work due to work-related accidents, occupational affections and common diseases [18]. Moreover, lifestyle habits and pathologic behaviours, such as alcohol consumption and tobacco smoking, that can lead to harmful diseases are more frequent in the seafarer population than in onshore populations [2]. Our study consequently looked both at the main causes of medical unfitness for work at sea among professional civilian seafarers and at the nosological distribution of these causes in an attempt to determine any specificity according to type of professional maritime practice (fishing, merchant shipping, shellfish farming or professional sailboating).

In regards to avoidable disabling diseases that could emerge in our study we also aimed to recommend preventive measures where possible.

In order to be as exhaustive as possible and to calculate an incidence rate of permanent unfitness for work at sea, the study was conducted on the entire population of French seafarers over more than a ten-year period.

## Methods

### Study type

This was a register study. In a retrospective approach, we conducted an investigation involving, on the one hand, an incidence study of all permanent medical unfitness for work at sea decisions taken in France over a given period and, on the other, an incidence study of the main reasons cited for these decisions. In addition to this nosological study, we carried out a sociodemographic study comprising a demographic description plus an account of the professional seafarer’s sector before they had become unfit. Thus the purposes of the study were first to determine the incidence rate of medical unfitness for work at sea among French seafarers, second to identify the conditions (diseases or accidents) causing such incapacity so as to set up prevention measures where possible and third to ascertain whether there were any overrepresentations of diseases according to category of unfit seafarers (fishers, merchant seafarers, shellfish farmers and professional sailors).

### Data collection

Information was collected from the computerised medical records of professional seafarers listed in the Aesculapius® database. Only SDs and the doctors at Toulouse’s Centre de Consultation Médicale Maritime (which is France’s TMAS (Telemedical Assistance Service) centre) had access to this database. The database listed all the French seafarers who had been seen by French SDs and, more importantly, all those referred to one of the four CMRAs between 2005 and 2016. It was housed in the Centre Administratif des Affaires Maritimes (administrative centre for maritime affairs) in Saint-Malo. There were 4978 files recorded in this database by the different CMRAs for the period in question. The CMRAs’ decisions concerning fitness for work at sea for each case (which were categorised as fit without restriction, fit with restrictions, temporarily unfit or permanently unfit) were also accessible. In addition, some of the decisions delivered by the different CMRAs involved in an appeal from the same seafarer were also described. Decisions concerning first registrations were also listed. The following data were collected:

- a unique identifier for the seafarer (which was not the seafarer’s registration number, thus ensuring anonymity) corresponding to their order of appearance in the CMRA files
- gender
- year of birth
- year in which the decision of permanent unfitness was taken by the CMRA
- CMRA location
- CMRA’s conclusion
- total time spent at sea and total time spent sea fishing, working on merchant ships, shellfish farming or professional sailboating prior to being declared unfit (this data can be found in the administrative section of the Aesculapius® database)
- ICD-10 coding of the main affection or the symptoms causing the unfitness. This ICD-10 coding activity was done by the SDs and entered in the seafarer’s medical file at the time of the medical examination and then transmitted to the CMRA.

The reference population was the entire population of French active seafarers/year. This annual number of French seafarers is available at the statistics department of the state-run social welfare institution for French seafarers: the Etablissement National des Invalides de la Marine (ENIM). In fact any achievement of a professional activity in the French maritime sector by a French seafarer is declared at ENIM. A national report is done every year by this welfare institution describing the total number of French seafarers who were registered (declared). They are classified by professional sectors (fishing, merchant shipping, professional sailboating and shellfish farming) as well.

### Study population

#### Inclusion criteria

The study population comprised all French professional civilian seafarers whose cases had been referred to the CMRA and who had received a final decision of permanently unfit for work at sea. The inclusion period was from 2005 to 2016 and concerned the entire French territory.

No distinction was made between the types of examinations (pre-employment, periodic, on request, return-to-work) initially carried out by the SD in relation to subsequent appeals to the CMRA.

In cases where several CMRAs had ruled for the same seafarer, only the final decision of medical unfitness was retained. Some applicants, when they disagreed with the final decision, had appealed to the National Medical College (*Le collège médical maritime national*), which had then requested a further assessment from yet another CMRA, meaning the same individual could be referred to a different CMRA several times.

#### Exclusion criteria

The following data were excluded:

- data from CMRAs prior to 2005 and after 2016 (collection stopped in 2017). We excluded data before 2005 because the information recorded prior to this was not sufficient for analysis. We chose 2016 as the cut-off point because the list of diseases included in the legislation changed in 2017. Hence, the same law references were used for the whole period of the study
- data from CMRAs declaring temporary fitness without restriction, temporary fitness with restrictions or temporary medical unfitness for work at sea
- data on professional seafarers placed on extended sick leave and for whom the decision on their fitness was therefore pending
- CMRAs’ decisions on applications to enter one of the seafaring professions. Some of these applicants appealed against a decision of unfitness for work at sea even though they had never boarded a boat before
- data concerning foreign seafarers
- data concerning naval seafarers, who belonged to a different governmental body.

**Fig. 1:**
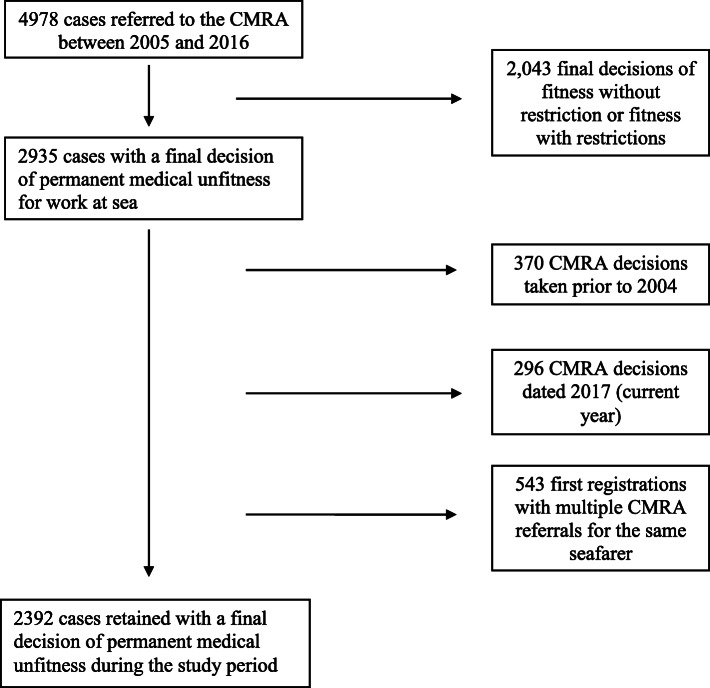
Flow chart showing the selection of files retained for the unfitness analysis

Between 2005 and 2016, 4978 professional seafarers’ files were referred to the CMRA. Of these 4978 cases, 2392 resulted in a decision of permanent medical unfitness for work at sea (Fig. 1).

### Main variables of interest

The main variables of interest were the number and age of seafarers per year who were declared unfit for work at sea in France. The different diseases or symptoms linked to the unfitness decisions (ICD-10 coding) was the secondary variable of interest. If two or more affections were potentially contributing to the cause of medical unfitness, only the main reason was retained, that is the affection entered first by the SD in the Aesculapius® database. Because of the large number of diseases and symptoms identified, the causes were grouped together by chapter of diseases as set out in the ICD-10. There are twenty-two (XXII) chapters in this 10th version of the ICD, but only fifteen of these were necessary to rank the diseases of the seafarers in our study. They were as follows:

- Neoplasms (chapter II)
- Endocrine, nutritional and metabolic diseases (chapter IV)
- Mental and behavioural disorders (chapter V)
- Diseases of the nervous system (chapter VI)
- Diseases of the eye and adnexa (chapter VII)
- Diseases of the ear and mastoid process (chapter VIII)
- Diseases of the circulatory system (chapter IX)
- Diseases of the respiratory system (chapter X)
- Diseases of the digestive system (chapter XI)
- Diseases of the skin and subcutaneous tissue (chapter XII)
- Diseases of the musculoskeletal system and connective tissue (chapter XIII), where many MSDs can be ranked
- Diseases of the genitourinary system (chapter XIV)
- Symptoms, signs and abnormal clinical and laboratory findings, not elsewhere classified (chapter XVIII)
- Injury, poisoning and certain other consequences of external causes (chapter XIX). Under this heading, we found polytrauma, burns, poisonings and all kinds of consequences of direct or indirect trauma suffered by professional seafarers. It should be noted that accidental injuries could affect the musculoskeletal system as well as other organs such as eye and brain injuries.

When it was relevant we also ranked diseases found as very numerous in ICD 10th sub-chapters. It thus permitted to compare some nosological groups of diseases leading to permanent unfitness for work at sea by categories of seafarers.

In all, we found 378 different diseases, injuries or symptoms cited for permanent medical unfitness for work at sea during the study period, which we ranked according to these fifteen chapters and according to four most frequent nosological groups (that is to say four ICD 10th sub-chapters).

### Other variables

Four different professional seafaring sectors were listed to cover every seafarer in France, that is to say fishing, merchant shipping, shellfish farming and professional sailboating. As we mentioned previously, the annual number of active seafarers was available in the national reports published by the ENIM. We read all the annual national reports published during the study period to find out the distribution of seafarers across each professional sector. We then combined these data with the data in the Aesculapius® database to categorise the professional sector of each unfit seafarer and to calculate the proportion of unfit seafarers among all seafarers for each sector. It was sometimes difficult to categorise the seafarers into one single professional sector. In these cases, we chose to classify them according to the sector (fishing, merchant shipping, shellfish farming or professional sailboating) that accounted for more than 50% of their career. This allowed us to determine whether any of the sectors had been more subject to unfitness decisions than others. We also wanted to find out if there was an overrepresentation of certain diseases in the different sectors.

Concerning the age of a French seafarer at the time of permanent unfitness for work at sea decision we ranked four major categories:

− 21 to 30 years old,

− 31 to 40 years old,

− 41 to 50 years old and − 51 to 60 years old. Then every age category has been split in two 5 year-period when it appeared to point out overrepresentation of cases.

In addition, total time spent at sea and total time spent sea fishing, working on merchant ships, shellfish farming or professional sailboating prior to be declared unfit were expressed in years.

### Analysis

In order to carry out the quantitative analysis of the data, we extracted the required information from the Aesculapius® database and transferred the coded elements into Excel® software. The incidence rate was calculated by dividing the annual number of cases concluded as permanently unfit for work at sea by the CMRAs by the annual number of registered active seafarers. We consequently calculated annual incidence rates of permanent unfitness for work at sea. Where data comparisons were necessary, we ran a Student’s t-test using the Statgraph® data analysis software. The significance threshold was set at *p* < 0.05. The null hypothesis assumed that no difference existed between the annual rates of unfitness for work at sea decisions concerning a particular disease. The sample size for establishing statistical significance was calculated at 30 subjects or more.

Among the four professional sectors of unfit seafarers (fishing, merchant shipping, shellfish farming or professional sailboating), we selected only those comprising more than 30 individuals for the purposes of statistical comparison. Two groups of seafarers were thus represented: fishers and merchant seafarers. The alternative hypothesis assumed that there was a difference between those two groups.

The 4 most frequent nosological groups at the origin of permanent unfitness were compared. The annual incidence rates for these 4 most frequent nosological groups were calculated by dividing the annual number of unfit fishers or merchant seafarers with the annual number of fishers or merchant seafarers registered nationally.

We also represented the total number of diseases, symptoms or injuries leading to permanent unfitness for work at sea at every age and age categories of our studied population.

Both sexes were analysed together in our representation of diseases.

## Results

### Sociodemographic study

**Fig. 2:**
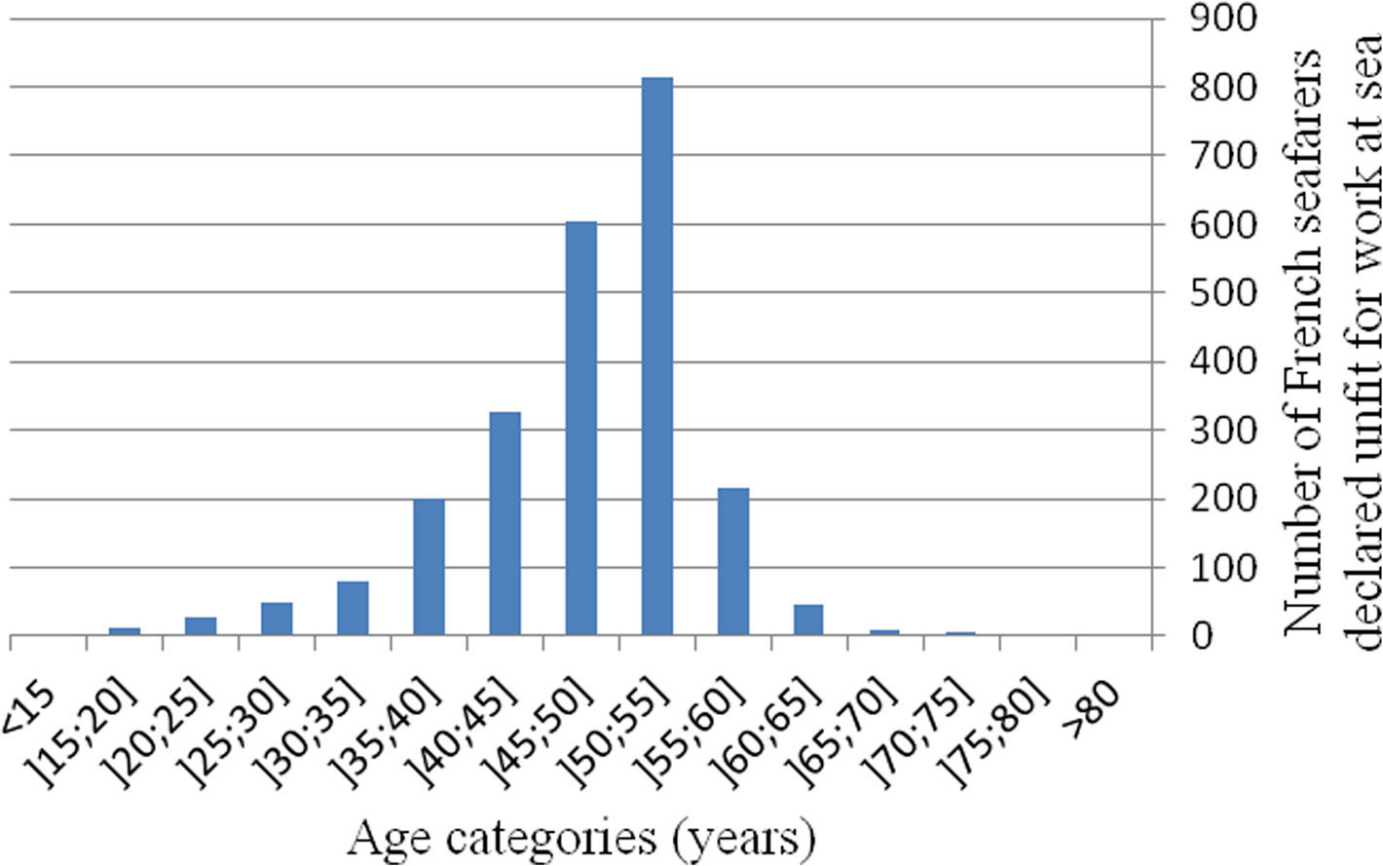
Age of French professional seafarers between 2005 and 2016 at the time they were declared permanently medically unfit for work at sea

The average age of the professional seafarers whose cases were concluded as medically unfit for work at sea by the French CMRAs between 2005 and 2016 was 48 ± 9 [15–81], with the most represented age group being 50–55 years (Fig. 2). More than 95% of those declared unfit were male.

An analysis of these cases showed an average time spent working at sea prior to unfitness of 186 months, or 15.5 years. The majority (56.6%) of the seafarers in the study had spent more than 15 years at sea before becoming unfit.

**Fig. 3:**
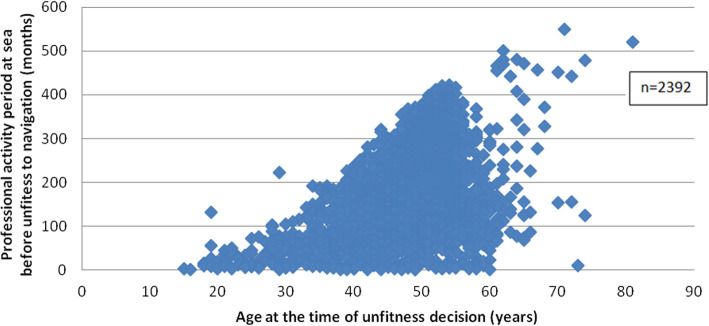
Total time spent working at sea in relation to age at the time of being declared permanently medically unfit for work at sea between 2005 and 2016.

**Fig. 4:**
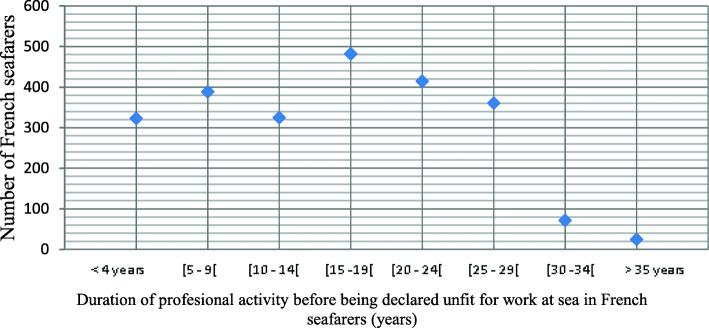
Duration of professional activity before being declared permanently medically unfit for work at sea during the study period

The distribution of the seafarers declared unfit is presented in Figs. 3 and 4.

The seafarers were often engaged in a combination of two or more of the four professional sectors. Professional sailboating and shellfish farming represented only a small proportion, however, of the population’s activities, with those spending more than 50% of their time either professional sailboating or shellfish farming accounting for only 1% each (17 and 27, respectively).

The fishing sector was clearly predominant among the professional seafarers declared permanently medically unfit for work at sea. We thus identified that our population was mainly composed of fishers and that these made up 67% of the total number of seafarers declared unfit, followed by merchant seafarers (31%) (Table 1).

**Table 1:** Number of French seafarers over the study period for each sector that represented 50% or more of their professional lives

> 50% of total activity	Fishing	Merchant shipping	Shellfish farming	Professional sailboating	Total
Number of French seafarers declared unfit for work at sea during the study period	1607 (67%)	741 (31%)	27 (1%)	17 (1%)	2392 (100%)

### Incidence study

#### Incidence of permanent medical unfitness for work at sea between 2005 and 2016

**Fig. 5:**
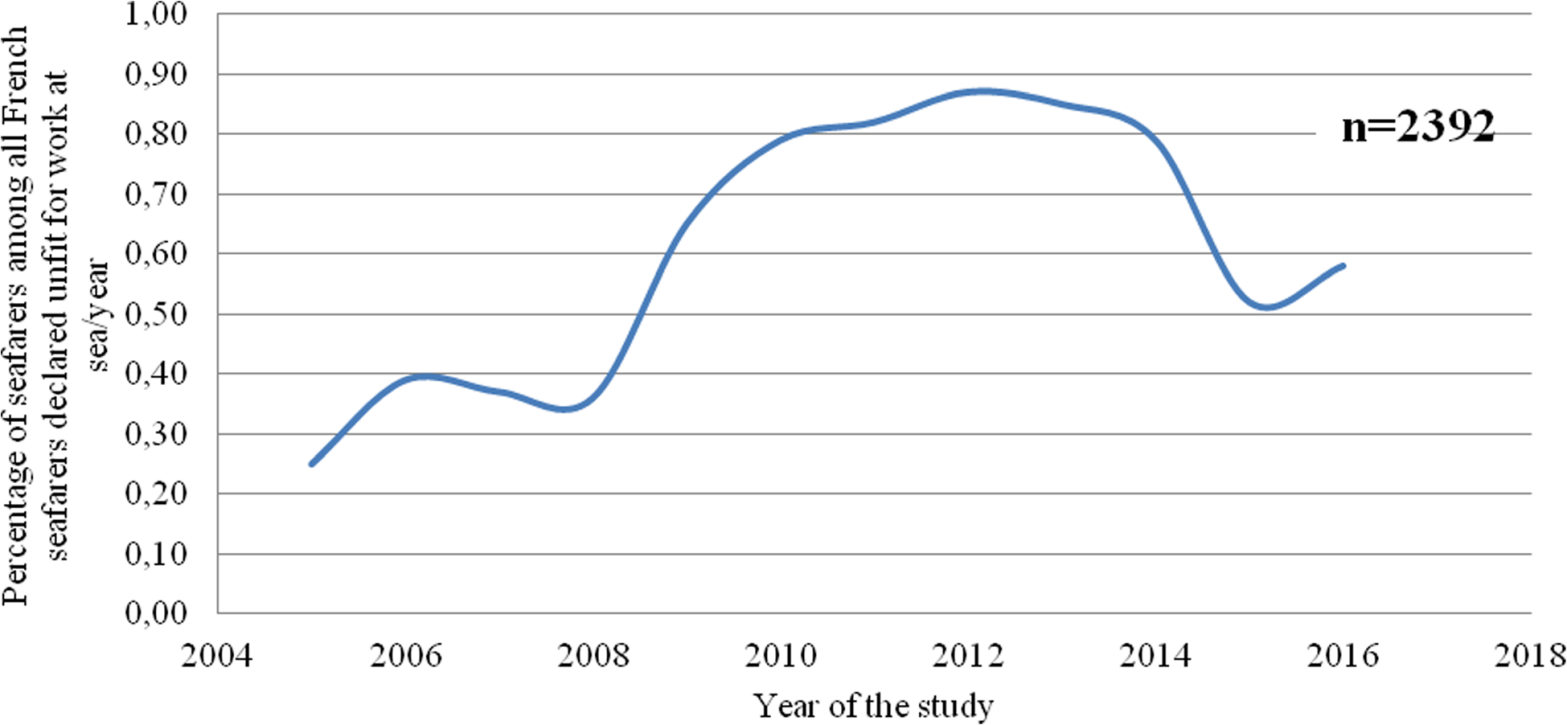
Incidence rate of medical unfitness for work at sea among French seafarers between 2005 and 2016

In Fig. 5 and Table 2, the headcount of seafarers declared permanently medically unfit is expressed in absolute numbers.

**Table 2:** Incidence rate in France of medical unfitness for work at sea over a 12-year study period

Year	Total number of seafarers with a decision of permanent medical unfitness for work at sea	Total number of French seafarers/year	Incidence rate
2005	97	39,051	0.24839313
2006	148	37,825	0.39127561
2007	134	36,351	0.3686281
2008	130	35,563	0.36554846
2009	225	34,232	0.65727974
2010	257	32,297	0.79573954
2011	248	30,017	0.82619849
2012	237	27,116	0.87402272
2013	234	27,404	0.85388994
2014	240	30,045	0.7988018
2015	211	40,300	0.5235732
2016	231	39,555	0.58399697

There was an overall increase from 0.25 to 0.58% in the incidence of permanent medical unfitness for work at sea across all French seafarers presenting at a medical fitness for work at sea examination between 2005 and 2016, with a drop in the rate of applicants being declared permanently medically unfit in 2015.

#### Causes of permanent medical unfitness between 2005 and 2016 by ICD-10 chapters of diseases and affections

The causes of permanent medical unfitness for work at sea listed according to chapters of the ICD-10 are shown in Table 3.

**Table 3:** Chapters of diseases leading to permanent unfitness for work at sea in French seafarers

Chapter of diseases	Total Number of unfit seafarers/chapter of diseases	Percentage
Diseases of the musculoskeletal system and connective tissue	573	23,95%
Injury, poisoning and certain other consequences of external causes	384	16,05%
Mental and behavioural disorders	324	13,54%
Diseases of the circulatory system	278	11,16%
Diseases of the nervous system	186	7,77%
Neoplasms	90	3,70%
Endocrine, nutritional and metabolic diseases	79	3,30%
Diseases of the digestive system	53	2,21%
Diseases of the ear and the mastoid process	41	1,70%
Symptoms, signs and abnormal clinical and laboratory findings, not elsewhere classified	35	1,46%
Diseases of the respiratory system	27	1,12%
Diseases of the eye and adnexa	25	1,04%
Diseases of the skin and the subcutaneous tissue	19	0,79%
Diseases of the skin and the subcutaneous tissue	11	0,45%
Unknown	267	11,16%

MSDs, which principally comprised chronic spinal injuries or diseases (in more than half of the cases) followed by lower limb and then upper limb affections, were clearly in the majority over the study period in question in terms of causing medical unfitness for work at sea.

Moreover, accidental injuries (the second cause of unfitness) were found to have frequently affected the musculoskeletal system, although other organs were also affected, including eye and brain injuries. The top 3 anatomical regions affected by these trauma were the upper limb (29% of the cases), the lower limb (27%) and the rachis (22%).

The affections were categorised as unknown in 267 of the CMRAs’ records (11.16%).

Ten of the diseases, injuries or symptoms causing medical unfitness in professional seafarers were preponderant. They were as follows: other intervertebral disc disorders M51 (*n* = 156), depressive episode F32 (*n* = 88), dorsalgia M54 (*n* = 60), recurrent depressive disorder F33 (*n* = 59), sprains and strains of the lumbar rachis and pelvis S33 (*n* = 52), mental and behavioural disorders due to use of alcohol (*n =* 52), acute myocardial infarction I21 (*n* = 49), shoulder lesions M75 (*n* = 47), epilepsy G40 (*n =* 47) and gonarthrosis M17 (*n* = 45).

The four most frequent affections leading to unfitness (given in decreasing order) according to age category were:

− 21 to 30 years old: accidental injuries, MSD, epilepsy and mental disorders.

− 31 to 40 years old: accidental injuries, MSD, mental disorders and Type I diabetes.

− 41 to 50 years old: MSD, accidental injuries, mental disorders and diseases of the circulatory system.

− 51 to 60 years old: MSD, accidental injuries, diseases of the circulatory system and mental disorders.

#### Comparison of the incidences among fishers and merchant seafarers of medical unfitness for work at sea for the 4 most frequent nosological groups

**Fig. 6:**
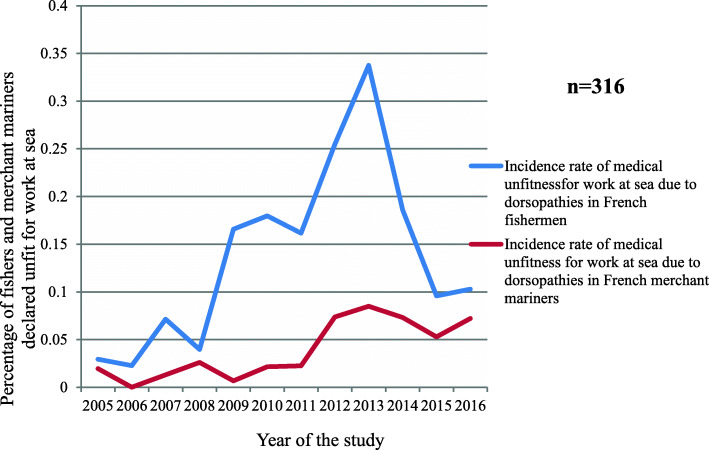
Incidence of dorsopathies in the French fishing and merchant shipping sectors leading to permanent medical unfitness for work at sea over the study period

Between 2005 and 2016, the incidence of medical unfitness linked to dorsopathies, that is to say M40 to M54 in the ICD-10 version (intervertebral disc injuries, dorsalgia, spondylarthrosis, cervical disc injuries, other dorsopathies, spondylopathies, ankylosing spondylitis, dorsopathies with deformities, symptomatic scoliosis, inflammatory spondylopathies), in the fishing sector was higher than that in the commercial sector (the difference was, however, only significant for the years 2009 to 2014 (*p* = 0.04)). In addition, there was a decrease in this incidence among fishers from 2013 onwards (Fig. 6).

**Fig. 7:**
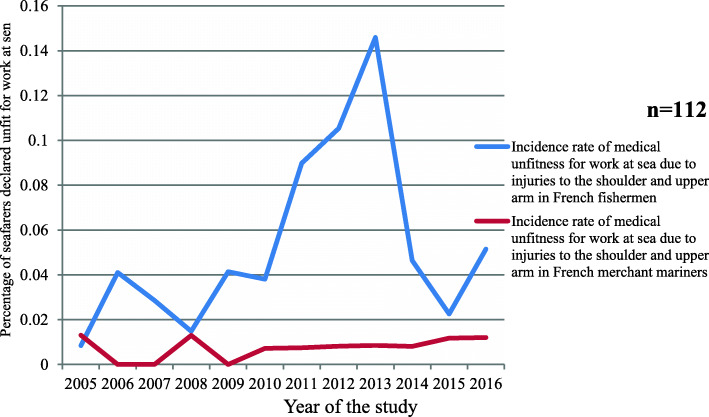
Incidence of injuries to the shoulder and the upper arm in the French fishing and merchant shipping sectors leading to permanent medical unfitness for work at sea over the study period

Between 2005 and 2016, the incidence of permanent unfitness linked to the consequences of injuries to the shoulder and upper arm (S40 to S49) in the fishing sector was higher than that in the commercial sector (*p =* 0.04 for the years 2009 to 2014) (Fig. 7).

**Fig. 8:**
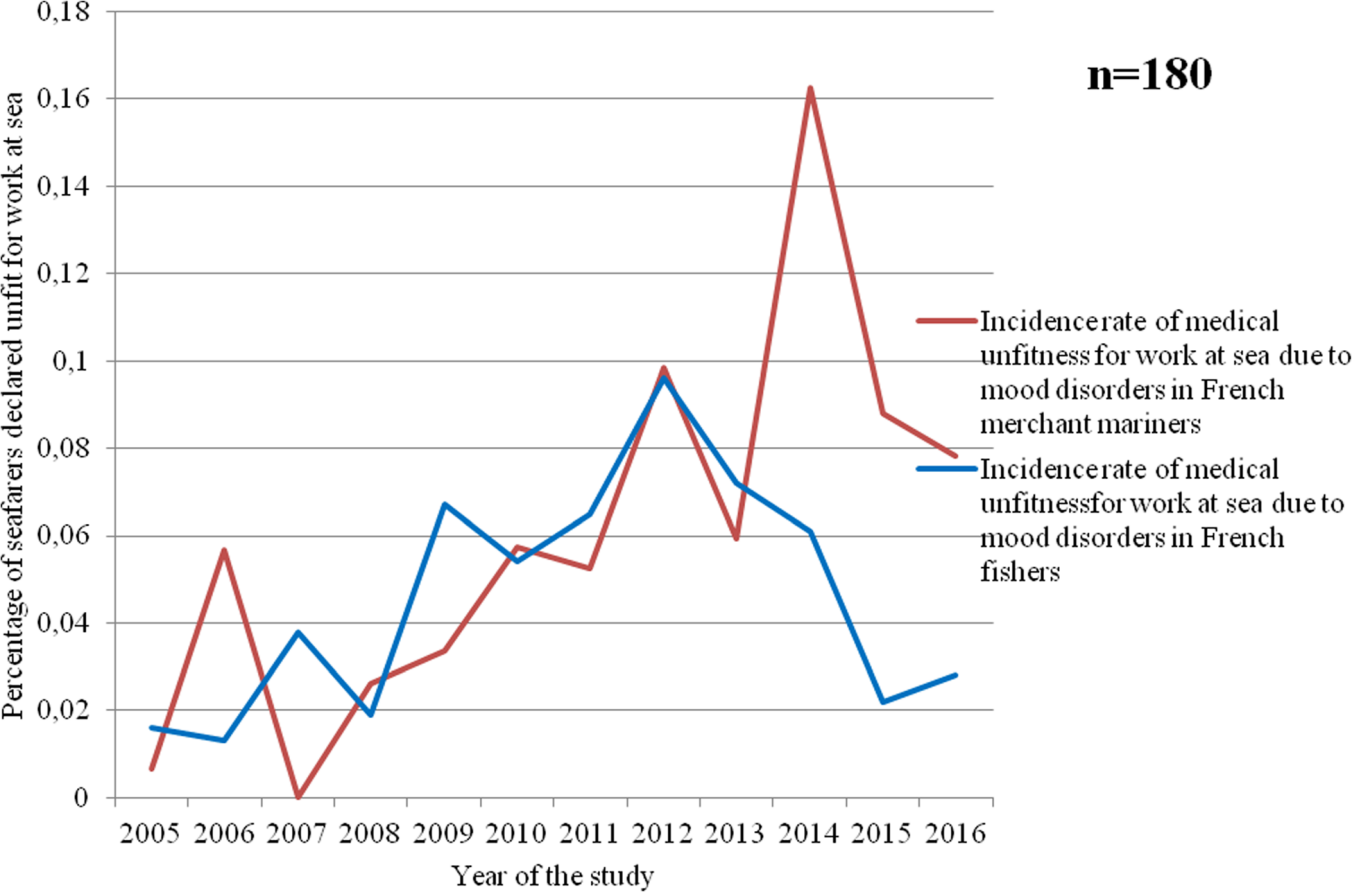
Incidence of mood disorders in the French fishing and merchant shipping sectors causing permanent medical unfitness for work at sea over the study period

Between 2005 and 2013, the incidences of mood disorders (F30 to F39) leading to unfitness were very similar in the fishing and merchant shipping sectors. However, from 2014 onwards, the incidence of mood disorders was higher among merchant mariners (*p* = 0.03) (Fig. 8).

**Fig. 9:**
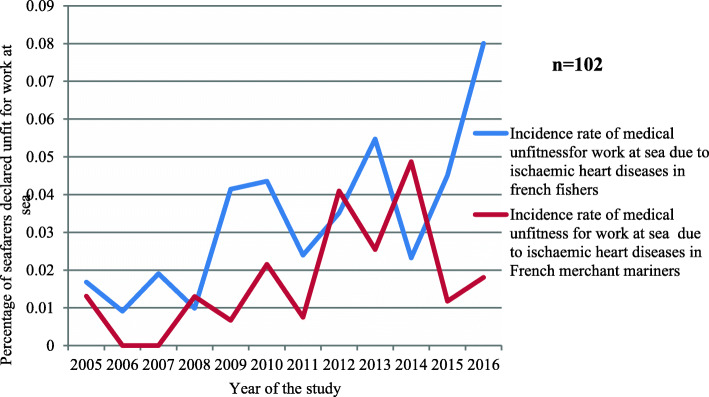
Incidence of ischaemic heart diseases leading to permanent medical unfitness for work at sea in French fishers and French merchant mariners over the study period

Between 2005 and 2015, the incidences of ischaemic heart diseases I20 to I25 (leading to medical incapacity for work at sea) were very similar in the fishing and merchant shipping sectors. From 2015 to 2016, however, there was a significant difference (*p =* 0.03) between the two, with a high incidence leading to unfitness among fishers and a low incidence among merchant mariners (Fig. 9).

## Discussion

In France, the incidence of permanent medical unfitness for work at sea in the population of professional seafarers as ruled by the CMRAs was found to have varied very little over the years. It remained at lower than 1% of the whole population of French seafarers presenting for a fitness for work at sea assessment. In other words, more than 99% of French seafarers were declared fit for work at sea. The incidence rate of unfitness tended to increase overall between 2005 and 2016, with a peak in 2012 and a drop in 2015.

Of the diseases and injuries found to lead to permanent unfitness for work at sea in France listed in the fifteen chapters, MSDs, which principally comprised chronic spinal injuries or diseases followed by lower limb and then upper limb affections, were predominant. The second most common were the consequences of different traumatisms, injuries or poisonings. The third most frequent cause was mental and behavioural disorders, including mood disorders and addictions, particularly alcoholism. Diseases of the circulatory system, namely coronaropathies, followed by diseases of the nervous system, primarily epilepsy, were also found to be common reasons for unfitness for work at sea. Neoplasms and endocrinal, nutritional and metabolic diseases, namely Type I diabetes, were less frequent. All the other diseases cited by the CMRAs (digestive, ENT, respiratory, ophthalmologic, dermatologic and genitourinary) were rare.

French fishers aged over 50 and with a 15-year career represented a high risk of unfitness. By contrast, young professional sailors and young shellfish farmers were very seldom declared unfit, but these were also very scarce in the population of French seafarers as a whole.

In the two younger age categories (21–40 years), unfitness was in many cases linked to diseases acquired in youth or young adulthood, such as epilepsy or Type I diabetes, whereas in the oldest age categories (41–60 years), these reasons tended to disappear and were replaced by degenerative diseases or illnesses linked to cumulative risk factors that induce disabling diseases, such as cardiovascular affections.

The incidence rate of permanent unfitness for work at sea varied little during the study period, remaining under 1%. A drop in the rate occurred in 2015. This was mainly due to a dilution effect caused by the entry in 2015 of 10,000 new professionally active seafarers (mainly in the fishing sector), almost all of whom were passed as medically fit without restriction for work at sea when they were recruited. Many of the seafarers (Figs. 2, 3 and 4) who were declared permanently medically unfit for work at sea had only been at sea for a very short time (a few months). This finding could be explained by an accidental injury that occurred at the beginning of their career and resulted in permanent medical unfitness for work at sea. As previously mentioned, it is difficult to compare this incidence rate with other incidence or prevalence surveys on maritime worker populations because the data collection methodologies, medical unfitness criteria and definitions of maritime activity, medical fitness and professional seafarer status differ from one country to the next [13].

MSDs were the predominant cause of permanent medical unfitness. This could be attributed to their disabling nature not just in everyday life but also in the performance of onboard tasks. Any functional loss of mobility leading to problems with standing, walking, gripping or balancing represents a hindrance to the seafarer because they risk falls or accidents caused by the vessel’s movements (rolling, pitching) or when using ladders, steps or onboard equipment [1, 17, 18]. By way of comparison, MSDs are the primary causes of permanent medical unfitness for work in almost all non-maritime sectors [3–5]. For example, in a study carried out on 6750 employees (in agriculture, construction, commerce, etc.) in the Loire region in 2004, MSDs were responsible for 44% of unfitness cases [19] compared with 24% in our study. In another study conducted in Brittany in 2009, MSDs accounted for 60% of unfitness decisions [20].

Decisions of medical unfitness for work at sea also concerned upper limb disabilities, especially the hands, with fishers being the largest group affected. This finding can be linked to data on the prevalence of maritime workplace trauma accidents involving hands among fishers in France [21] and worldwide [16–18]. The professional prognosis of these accidents is therefore serious. Corroborating these notions is the fact that there were more injuries leading to medical unfitness among professional seafarers than among private-sector employees. In a comparable study carried out in the field of land-based occupational health, the rate of injury, poisoning and certain other consequences of external causes of unfitness was substantially lower at only 2% [19] than the 16% in our study. This also appears paradoxical, however, because this same maritime work was to a large extent responsible for the affections that forced seafarers to give up their professional activities [22–25].

Chronic illnesses such as mental and behavioural disorders and diseases of the circulatory system were recorded as only the third and fourth most common causes of permanent medical unfitness for work at sea. However, we had expected these to be top of the list because not only are mental disorders and ischaemic heart diseases frequent among professional seafarers but they are sometimes very difficult to treat at sea at the time of their decompensation [15]. Nevertheless our figures are consistent with studies conducted on land-based occupational health, where mental and behavioural disorders are cited in 21% of unfitness decisions, half of which are due to a mood disorder [19, 20].

Onboard working conditions and professional tasks are known to put a strain on the circulatory system. Indeed, SDs had to take this factor into account along with remoteness from land-based care in the event of decompensation on board in their decisions on cases of ischaemic heart diseases. A seafarer would not receive the same essential effective, monitored treatments at sea that they would in hospital [10, 11, 18, 26]. It is therefore specified in the decree of 3 August 2017 that affections of the circulatory system are not compatible with working at sea. As a comparison, diseases of the circulatory system are responsible for 5 to 7% of land-based permanent unfitness to work in France (although this proportion rises to 13% for occupations in the construction and driving sectors [19, 20]) versus 11% in our study.

Occupational health studies conducted in non-maritime sectors have found similar age averages for permanent medical unfitness for work. For example, a study of 1052 unfitness cases in Brittany in 2009 showed an average age of 46 [20]. Further, a study conducted in the Pays de Loire between 2002 and 2004 reported an average age of 45 with a peak in unfitness decisions for those aged between 55 and 59 [19]. According to both these studies, professional wear and tear and the demographic characteristics of the populations studied were the main risk factors of medical unfitness.

It is possible that 15 years of professional activity in the maritime sector involves sufficiently harsh working conditions to lead to affections that are disabling as far as working at sea is concerned. In France, seafarers have their own retirement organisation and can apply for different pensions linked to the period they have spent working as a seafarer. One of the most important eligibility conditions for an early retirement pension is the requirement of 15 years of service. This figure was confirmed by the SSGM’s regional study of total permanent unfitness for work at sea decisions conducted between 2009 and 2010 [27]. It found that the 15-year mark effectively corresponded to a frequent trigger point for unfitness decisions. However, we were not able to identify many cases of medical unfitness among older seafarers. It could be that those with health problems who were close to retirement age may have chosen to end their careers in a different way from being declared unfit. Professional seafarers nearing the end of their careers who could potentially have been declared permanently medically unfit for work at sea may have found it more socially advantageous to take early retirement. Professional seafarers with less than 15 years of service may also have accumulated multiple sick leaves and waited for retirement rather than initiating the unfitness test process with the SD.

The regional study cited above identified approximately the same distributions of professional seafarers declared unfit for work at sea, that is to say 65.26% in the fishing sector, 21.6% in merchant shipping and 5.79% in shellfish farming [19].

This predominance of the fishing sector could be explained by the fact that the greatest number of professional seafarers worked in this sector. The low incidence of medical unfitness within the shellfish farming sector was undoubtedly due to the specificity of this activity and the low number of people working in the sector. We understand that there were fewer cases of permanent medical unfitness for work at sea in shellfish farming, where working in the open sea is rare and remoteness from land is therefore minimal, because redeployment is easier within this sector than it is in the fishing sector.

Limitations:

Our study had some limitations. First, the collection of information was incomplete because 11% of causes were unknown or potentially misclassified. In addition, the fact that the disease codes had been entered by different SDs, who had no protocol and who based their decisions solely on the ICD-10’s dictionary of specialised terms, meant there was an inherent risk of potentially different interpretations. This could without doubt have influenced the results by over- or under-representing one particular affection.

One major bias resulted from the fact that that an individual’s health status was limited to one code. There was thus an interpretation bias in the data because some affections were closely related. For example, the consequences of chronic alcohol poisoning could be coded under F10 ‘Mental and behavioural disorders due to use of alcohol’, F32 ‘Depressive episode’, F33 ‘Recurrent depressive disorder’, K70 ‘Alcoholic liver disease’, K74 ‘Fibrosis and cirrhosis of liver’, and so on. Hence, the SDs’ choices were inevitably arbitrary.

It was also difficult to clearly define the seafarers’ role and type of activity. Professions can evolve over the course of a career, with different positions and different activities taken up. For example, a deckhand in the fishing sector might become a mate, skipper and then owner. Since crews are kept to a minimum for economic reasons, skippers may also undertake watchkeeping duties (even though they are theoretically exempt) and owners may work on board as deckhands.

In addition, decisions of permanent unfitness for work at sea affected mainly professional seafarers over the age of 50. If professional careers in this sector were to be extended, it is highly likely this would result in an increasing number of unfitness decisions in the future, which is in line with the increase observed in this respect between 2004 and 2016. Nevertheless we did not realised an age adjusted analysis that could highlight the evolution of diseases by age categories in the French seafarers population. We can only suppose that some degenerative diseases (most of all degenerative MSD) occur earlier in our studied population than in others working population. Therefore the role of maritime work factors in the appearance of such diseases needs to be assessed in further studies.

Finally, while the system of collecting medical information using the Aesculapius® database meant a large amount of data could be gathered, the healthy worker effect and the self-selection effect were highly likely. Seafarers with disabling affections may have ended their maritime activity of their own volition without going through the medical unfitness process. In this case, the population would therefore comprise a large majority of ‘healthy’ people.

### Recommendations

While unfitness decisions remain rare across the whole population of French professional seafarers, the medical and social prognosis of these incapacities is poor [1, 2, 6–10]. However, in many cases, it seems that the health problems at the origin of these medical unfitness decisions can be subject to primary and secondary prevention measures. First, the high incidence of MSDs should prompt initiatives to combat all the risk factors that generate them. These could include biomechanical, psychosocial and individual constraints, such as sedentary lifestyles, obesity and Type II diabetes, which are still very frequent among seafarers [22–25]. Because onboard biomechanical and psychosocial demands are high, targeted ergonomic and organisational measures must be put in place to facilitate handling, porting loads and the movement and transfer done by crew members.

In addition, reducing psychosocial constraints by promoting work arrangements that incorporate a high degree of decision-making latitude, social support, sympathetic management and a listening and communication protocol for everyone on board can have an impact not just on MSDs but on anxiety and depressive syndromes, which unfortunately account for a large proportion of unfitness decisions [6, 18].

Continued onboard safety improvements will also help to reduce maritime accidents at work [16–18, 22] and, given the prominence of the sequelae of trauma figuring among the reasons for unfitness, also therefore the incidence of unfitness due to accidents.

Finally, an ongoing promotion of a healthy lifestyle among seafarers (e.g. by reducing addiction [27, 28] and encouraging regular physical activity and a diet containing less fat, salt and sugar) would help to reduce the high proportion of metabolic disorders and in particular cardiovascular diseases [11, 26], which are still very common among the illnesses causing unfitness.

## Conclusion

A decision of permanent medical unfitness for work at sea results either from the seafarer’s inability to continue working on board without the risk of a deterioration in their state of health or from a medically observed mismatch between the seafarer’s physical and/or psychological health and their own or their crew’s safety on board.

Fortunately, the incidence rate of permanent unfitness for work at sea was low (less than 1% of French seafarers were subject to this decision). The average age of the professional seafarers whose cases were concluded as medically unfit for work at sea was 48 ± 9 [15–81], with the most represented age group being 50–55 years. More than 95% of those declared unfit were male.

The majority (56.6%) of the seafarers had spent more than 15 years at sea before becoming unfit. The fishing sector was clearly predominant among the professional seafarers declared permanently medically unfit for work at sea.

The main causes of unfitness, namely MSDs, injuries involving the rachis or limbs, mental disorders and diseases of the circulatory system, are all non-communicable diseases with prevention opportunities.

Measures targeted at personal habits, for example diet and drug or alcohol consumption, and at ensuring safety in onboard physical activities and working conditions could improve prevention and limit the main causes of medical unfitness. The social and financial impact of these actions could be felt in just a few years.

Further studies on the effectiveness of such campaigns with regard to the health status of seafarers and the incidence of medical unfitness could be relevant for all stakeholders and for identifying best practice.

Finally, there is very little difference between the professionally active French sea-based and land-based populations. The affections responsible for unfitness for work were approximately the same in both contexts, however the distributions were different, with a higher incidence of medical unfitness decisions related to injuries, addictions and ischaemic heart diseases in sea-based than in land-based work environments.

Further studies are also needed to determine precisely if, as we suppose, most of the MSDs, accidents, injuries and mood disorders at the origin of unfitness for work at sea decisions are indeed work-related.

## Data Availability

All data generated or analysed during this study are included in this published article.
